# How Does CEO’s Environmental Awareness Affect Technological Innovation?

**DOI:** 10.3390/ijerph16020261

**Published:** 2019-01-17

**Authors:** Qinghua Huang, Xiding Chen, Mi Zhou, Xiaoqin Zhang, Lingling Duan

**Affiliations:** 1 School of Economics and Management, Southwest University, Chongqing 400715, China; hqh@swu.edu.cn (Q.H.); z19951001@email.swu.edu.cn (M.Z.); zxq0113@email.swu.edu.cn (X.Z.); 2 Department of Finance, Wenzhou Business College, Wenzhou 325035, Zhejiang, China

**Keywords:** CEO, environmental awareness, technological innovation

## Abstract

In this paper, we conduct an empirical study on the impact of CEOs’ environmental awareness on technological innovation. To this end, we obtain a large sample with 7615 observations from Chinese A-share listed firms between the years of 2003 and 2014. Our empirical results show that a CEO’s environmental awareness has a significant positive impact on technological innovation of his/her enterprise. Environmentally conscious CEOs will invest more in R&D and obtain more patents. This will help their enterprises achieve higher efficiency of technological innovation. Furthermore, the environmental awareness of a CEO has a more significant impact on technological innovation if his/her enterprise is subject to a higher level of monitoring. We also conduct robustness check of our findings and provide managerial insights and proactive government policies to raise the environmental awareness of CEOs and improve the innovation vitality of enterprises.

## 1. Introduction

It is generally believed that technological innovation is an important approach for an enterprise for building its core competitive advantage. A number of existing studies explore important factors affecting technological innovation, such as ownership structure [1], stock liquidity [2], the number of analysts [3], CEO tenure [4,5], and CEO incentive mechanism [6,7]. These studies provide a range of managerial insights for businesses to enhance their innovative capability. This paper aims to further enrich this literature by focusing on CEO’s awareness of environmental protection.

In a typical enterprise’s management structure, CEOs tend to be at the very top, and their decisions are closely related to the company’s strengths, weaknesses and needs [8]. For example, if an enterprise is led by a CEO with longer tenure, it has more time to conduct extensive technology search, acquire preferred R&D skills, and hire more R&D personnel to implement exploratory innovation strategies [4,5,9]. CEO’s leadership style is also an important factor affecting the company’s technological innovation. For example, a transformational CEO can have a significant lifting effect on promoting product innovation [10]. There is also research on how a CEO’s focus on the past, present and future will affect the development speed of new products. For example, in a stable environment, if a company’s CEO pays more attention to the past and the present, but less attention to the future, the company’s new product development speed will be higher. On the other hand, in a dynamic environment, a company’s CEO needs to pay less attention to the past, but more attention to the present and the future, in order to achieve faster new product development [11].

Therefore, it can be concluded that the influence of CEO on corporate innovation is indeed significant, and this effect is manifested through the multi-faceted qualities of the CEO. One of the most important qualities is CEO’s environmental awareness, which reflects people’s views about the human–nature relationship, develops within the context of human societies and affect human experience and behavior [12]. However, there is little research on the role of CEO’s environmental awareness in enterprise technology innovation. In this paper, we make a major research contribution by filling this research gap.

According to Upper Echelon Theory [13], the strategic choice of a company is, to a large extent, influenced by personal cognition of its CEO. As a concrete manifestation of CEO’s cognition, can CEO’s environmental awareness impact the strategic decisions of their enterprises, including technological innovation? what will happen to the relationship between the CEO’s environment awareness and technological innovation at different levels of monitoring? There has been much literature on the significance of CEO’s environmental awareness in enterprise management. Some scholars propose that managers’ environmental awareness is an important factor affecting the implementation of enterprise environmental strategy. Sharma [14] finds that managers’ interpretation of environmental issues significantly affects enterprises’ environmental strategies. The results show that managers are more likely to adopt forward-looking environmental strategies when they regard environmental problems as opportunities for enterprises’ development. Aragón-Correa et al. [15] argue that if more managers in an organization think that enterprises should take on environmental responsibility, a high level of executive environmental commitment will appear to promote the development of environmental protection and the implementation of forward-looking environmental strategies. Roxas and Coetzer [16] find that if managers believe that the institutional environment can support the implementation of environmental policies, they are more likely to have a positive attitude toward environmental issues. Zhang et al. [17] support the view that environmental awareness of senior executives plays a crucial role in external normative pressures and in the practice of energy conservation policies. At the same time, several studies explore the impact of managers’ environmental awareness on corporate green innovation. Egri and Herman [18] consider that environmentally conscious executives are good at capitalizing on the importance consumers, suppliers and competitors attach to green innovation. They actively develop new products to meet consumer requirements and to gain competitive advantages. Burki and Dahstrom [19] confirm that top management commitment to sustainability yields higher levels of green process innovation, green managerial innovation, and customer cooperation. Gadenne et al. [20] argue that executives will adopt green innovations to upgrade their environmental outputs if they realize that environmentally friendly business policies will bring beefier profits.

Although the existing research helps us understand how the environmental awareness of managers triggers green innovation, there are still several limitations. First, there is very little research on the environmental awareness of CEOs and how it relates to technological innovation in their enterprises. Second, enterprise technology innovation is a long-return and high-risk endeavor. The premise that CEO environmental awareness affects technology innovation is that enterprises can effectively reduce the moral hazard in the principal-agent relationship so that CEOs commit to the long-term development of their enterprises. However, a CEO may take advantage of information asymmetry and pursue his/her own self-interest. Hence, monitoring may play an important role between a CEO’s environmental awareness and the technological innovation of his/her enterprise.

In view of the above discussion, in this paper, we study the two key factors of CEO’s environmental awareness and the level of monitoring. We collect sample data from Chinese A-share listed firms during the years of 2003–2014, integrate Upper Echelon Theory and Principal-Agent Theory, and investigate the relationship between CEO’s environmental awareness and enterprise technology innovation and the moderating role of monitoring level.

## 2. Research Methodology

### 2.1. Research Hypothesis

#### 2.1.1. CEO’s Environmental Awareness and Technological Innovation

The attitude of CEO will significantly affect the scope and speed of enterprises’ response to environmental issues. Only when CEOs are fully aware of the importance of environmental innovation, will they view it from a strategic perspective and invest for its implementation [21]. According to Porter Hypothesis [22], proper environmental regulation can promote innovative activities from enterprises. These innovations will improve productivity of enterprises, thus offset expense of environmental protection and enhance the profitability. Therefore, CEO’s stronger awareness of environmental protection may be similar to environmental supervision and has similar positive effect on enterprise innovation.

Specifically, on one hand, CEOs with stronger environmental awareness can overcome the cost pressure of green development through technological innovation. Most of the industries in developing countries are at the low end of the global value chain, and their products are characterized by low added value and high pollution emissions. Affected by this development model, developing countries are caught in a dilemma. When the economy is stable, pollutant emissions inevitably increase and environmental quality continues to deteriorate. When promoting environmental protection and pollution control, as the cost of products increases and the competitiveness of exports declines, it will inevitably lead to a decrease in economic growth. In such a dilemma, technological innovation is crucial to driving the company’s upgrade. Therefore, in developing countries, CEOs with stronger environmental awareness can more clearly understand the cost pressure brought by development of environmental strategy. Hence, in the process, they will actively promote innovations to increase the chance for success. On the other hand, CEOs with a higher level of environmental awareness can more clearly identify the potential benefits that environmental protection can bring to companies. They can make full use of stakeholders’ attention to environmental strategy, develop new products to meet customer requirements, share innovation risks with suppliers, focus on competitors’ strategies to expand market share, and ultimately form a unique competitive advantage [18]. Therefore, CEOs with higher levels of environmental awareness are more motivated to adopt technological innovation to achieve green innovation and better environmental performance. In addition, CEOs’ environmental awareness as a specific manifestation of his cognitive characteristics means his recognition of sustainable development. Technological innovation is an important solution to the problem of sustainable development [23], so the environmental awareness of CEOs is also more likely to promote non-environment-related technological innovation. Based on above analysis, we propose the first hypothesis H1:

**Hypothesis** **1a** **(H1a).** *Ceteris paribus, if a firm’s CEO has stronger environmental awareness, the firm will invest more in R&D*.

**Hypothesis** **1b** **(H1b).** *Ceteris paribus, if a firm’s CEO has stronger environmental consciousness, the firm will generate more R&D output*.

**Hypothesis** **1c** **(H1c).** *Ceteris paribus, if a firm’s CEO has stronger environmental awareness, the firm will have higher efficiency in R&D*.

#### 2.1.2. The Role of Monitoring

We hypothesize that the positive impact of a CEO’s environmental awareness on technological innovation is more pronounced in an environment with higher level of monitoring. On the one hand, such an environment has a preventive effect on the CEO’s opportunistic behavior. When enterprises have greater internal and external monitoring, they can effectively reduce the agency costs and achieve effective corporate governance [24,25]. This is especially true in developing countries such as China, where many state-owned enterprises have a large room for opportunistic behavior because of the unclear nature of the exact ownership and the unclear relationship between rights and responsibilities. CEOs of non-state-owned enterprises may be more willing to adopt technological innovations that are consistent with corporate interests in an environment with more monitoring. Moreover, as a key market intermediary, analysts are the main information producers in the capital market and can mitigate information asymmetry [26]. They can help companies better understand the value of long-term capital investment, and thus help CEOs identify and select more innovative projects.

On the other hand, an environment with more monitoring inside and outside an enterprise can promote its technological innovation. First, when the company’s monitoring is strong, it indicates that the company has effective corporate governance. Second, due to social responsibility and ethical considerations, environmentally conscious CEOs will be more active in implementing technological innovation activities to meet investors’ environmental preferences. Rational institutional investors have economies of scale in information acquisition. They can identify and invest in companies that are inherently innovative. This also makes a company’s CEO more motivated to implement technological innovation strategies [27]. At the same time, institutional investors are more “locked in” to an enterprise they invest in because of their high shareholding ratio. As a result, they tend to encourage enterprises to innovate [28]. Kim [29] found that external regulators such as institutional investors or analysts can participate in corporate governance through their own expertise and information resources. They can urge CEOs to carry out technological innovation and focus on long-term research and development. Based on the above discussions, we propose the second hypothesis as follows:

**Hypothesis** **2** **(H2).** *Ceteris paribus, the positive impact of a CEO’s environmental awareness on technological innovation is more pronounced in enterprises facing higher level of monitoring.*

### 2.2. Data and Samples

In this paper, we use a large sample of Chinese A-share listed firms (A-shares are ordinary shares of the RMB. They are ordinary shares issued by domestic companies for domestic institutions, organizations or individuals to subscribe and trade in RMB. B-shares are special stocks of the renminbi. It is a foreign share listed and traded on the stock exchanges in China (Shanghai, Shenzhen) in the form of RMB denominations, foreign currency subscriptions and purchases.) during the years of 2003–2014. Excluding B-share, firms with negative book value and total assets, financial industry observation, and any missing variables, we obtain a final sample size of 7615. Among the variables, the R&D input data and patent data of listed companies are collected manually. R&D input data are extracted from the annual reports of listed companies. On the other hand, patent data are collected through the China Patent Announcement Query System of the State Intellectual Property Office because China’s financial disclosure system does not require listed companies to disclose R&D investment and patent data. In addition, we also use the financial data and stock return data of Listed Companies in Shanghai and Shenzhen, which are supported by China Stock Market Research Database (CSMAR) of Guotaian Information Technology Corporation.

### 2.3. Variable Measurement

#### 2.3.1. Technological Innovation of Enterprises

Existing literature has not reached a consensus on the measurement of enterprise technological innovation. It can be mainly divided into the following three measurement methods. The first one is to measure enterprise technological innovation through R&D investment, which includes labor and financial input. The second one is to measure technological innovation by R&D output, which can be measured by the number of new products and patents. The third one can be measured by both by innovations and their productions from the perspective of R&D input and R&D output. We assume that the impact of CEO’s environmental awareness on technological innovation will not only increase technological innovation, but also increase the number of technological innovation productions. Therefore, this paper uses the two indicators of R&D input and R&D output to measure technological innovation of enterprises. We use the ratio of total R&D expenditure to total assets lagging behind one period to measure the R&D investment of an enterprise in that year. If the information of total R&D expenditure is missing, we assign it to 0. In addition, we measure the R&D output of an enterprise by its patents published in the year.

#### 2.3.2. CEO’s Environmental Awareness

Based on Kollmuss and Agyeman [30], Gifford and Nilsson [31], Meyer [32], Casaló and Escario [33], personal background can reflect the level of environmental awareness. The higher the education level is, the stronger the awareness of environmental protection will be. Environmental awareness of young people is stronger than that of the elderly and environmental awareness of people whose majors are environment-related is stronger than people majoring in other disciplines. Therefore, this paper chooses the three variables of education level, age and whether they are environmental majors to measure a CEO’s environmental awareness. A CEO’s educational level is divided according to their educational level. The CEOs, whose educational level is lower than the undergraduate degree, are given a “0” value, and the CEOs whose educational level is higher than the undergraduate degree are given a “1”. Age was divided into two quantiles: the younger group was assigned a value of “1” and the older group was assigned a value of “0”. The CEO whose college or higher education major is environmental science, environmental engineering, environmental management and other environmentally related specialties are given a “1” value, and the others are assigned a value of “0”.

#### 2.3.3. Monitoring

Based on the literature on corporate monitoring, we select three representative indicators: the nature of ownership to measure the internal monitoring intensity, the proportion of institutional investors and the number of analysts to measure the external monitoring intensity.

In addition, following existing studies [34,35], we select the following control variables: the natural logarithm of sales revenue (Sales), the ratio of PPE (Property, plant, and equipment) over the total assets (PPE), the stock return (Return), the enterprise scale (Size), the growth opportunities of companies (MTB), the growth rate of sales revenue (Sales Growth), the return on assets (ROA), the leverage ratio of companies (Leverage) and the case flow of companies (CFO). The definitions of the above variables are listed in Table 1 below.

### 2.4. Descriptive Statistics

We calculate the descriptive statistics of CEOs’ environmental awareness and find that the average value of 1.038 and the median value of 1. It indicates that many CEOs have exactly one characteristic from higher education, younger age and environmental professional background at the same time. Next, we classify companies with Environment greater than or equal to 2 as a group of high-environment-conscious CEOs, and those with Environment less than or equal to 1 as a group of low-environment-conscious CEOs, and then perform descriptive statistical analysis on the variables in the regression model. As shown in Table 2, we can see that the average value of R&D_t+1_ and Patent1_t+1_ of the high environmental awareness CEO group is larger than that of the low environmental awareness CEO group, but the average value of Patent2t+1 is lower than the low environmental awareness CEO group. Thus, it cannot be seen how the CEO’s environmental awareness affects the level of technological innovation of enterprises. From the two perspectives of Sales_t_ and Size_t_, the sales revenues and company sizes associated with CEOs with high environmental awareness are slightly larger than those associated with the low environmental awareness CEOs. However, the growth rate of sales revenue is slightly lower than that of the low environmental awareness CEO. In addition, from the perspective of monitoring level, the average value of the three variables of the high environmental awareness CEOs, state, Ins, and Analyst, is higher, which indicates that enterprises with higher monitoring level are more likely to hire CEOs with high environmental awareness. Since descriptive statistics can only provide a preliminary understanding of the data, we also need to use more rigorous empirical analysis.

## 3. Empirical Analysis

### 3.1. Correlation Analysis

Table 3 reports the test results of the correlation coefficients between variables. Overall, it can be seen that the correlation coefficient between the explanatory variables is basically no more than 0.4. This indicates that there is no serious multicollinearity problem between the explanatory variables, and it will not have a greater impact on the regression results. Moreover, there is a significant positive correlation between the CEO’s environmental awareness and the number of published invention patents and R&D efficiency. There is a positive correlation between the CEO’s environmental awareness and R&D expenditure of the enterprise, but it is not significant. The above results support the hypothesis that a CEO’s environmental awareness can promote the technological innovation of his/her enterprise. However, the coefficients and saliency in the correlation coefficient matrix considers only the relationship between a single explanatory variable and the dependent variable. Nonetheless, a company’s technological innovation is the result of a combination of factors, so we therefore use regression methods to further test the relationship between CEO’s environmental awareness and corporate technological innovation.

### 3.2. CEO’s Environmental Awareness and Technological Innovation of Enterprises

This paper analyzes the data through the Stata software and uses the following regression equation to test the impact of CEO’s environmental awareness on technological innovation:

(1) $${\mathrm{Innovation}}_{t+1}=\alpha_{0}+\alpha_{1}{\mathrm{Environment}}_{t}+\sum_{k}\alpha_{k}{\mathrm{Controls}}_{t}^{k}+\varepsilon_{t}$$

Among them, Innovation_t+1_ represents the index of technological innovation of enterprises. In order to avoid the influence of other factors on the relationship between environmental awareness and technological innovation, we also set some controls in the regression equation. The regression results of Model 1 are shown in Table 4:

Firstly, according to Table 4, the test results in column (1) support hypothesis H1a. When R&D_t+1_ is a dependent variable, the coefficient of Environment is positive (0.068) and significant at 5%. It indicates that CEO’s environmental awareness is positively correlated with R&D investment and enterprises with CEOs with stronger environmental awareness have higher R&D investment. By observing the coefficients of control variables, we find that Sales, Return, MTB, ROA and CFO are positively correlated with the next R&D investment, while PPE, Size and Leverage are negatively correlated with the next R&D investment. Secondly, the test results in column (2) support the H1b. When Patentl_t+1_ is a dependent variable, the coefficient of Environment is positive (0.124) and significant at 1%. It indicates that a CEO’s environmental awareness is significantly positively correlated with the number of patents issued and CEO’s environmental awareness can improve R&D output. Thirdly, the test results in column (3) support the hypothesis H1c. That means that CEO’s environmental awareness can improve R&D efficiency of enterprises. In order to test the influence of CEO’s environmental awareness and R&D efficiency in H1c, we control the current R&D investment on the right side of the regression equation according following Hirshleifer et al. [24]. Then we use Patent1_t+1_ as a dependent variable, and the coefficient of Environment is still positive (0.112). At the level of 1%, it shows that under the control of current R&D investment, CEO’s environmental awareness is still significantly positively correlated with the number of invention patents issued. Under certain circumstances of R&D investment, CEO’s environmental awareness is positively related to R&D output. Therefore, the test results in Table 3 confirm H1, indicating that CEO’s environmental awareness has a positive impact on R&D input, R&D output and R&D efficiency. These results confirm that CEOs with high environmental awareness can significantly promote technological innovation.

### 3.3. The Role of Monitoring

In order to verify the H2, this paper introduces the monitoring variables based on Model 1. They include the type of ownership, the proportion of institutional investors and the number of analysts. Among them, the ownership is used to measure the internal monitoring of enterprises. The proportion of institutional investors and the number of analysts are used to measure the intensity of external monitoring.

#### 3.3.1. Ownership Type

The internal monitoring of enterprises can be significantly different because of the different ownership types. The principal-agent relationship of state-owned enterprises is characterized by many levels of principal-agent, long chain, vacant owner and double roles of intermediaries at all levels, which makes the internal monitoring of state-owned enterprises weaker than that of private enterprises. According to H2, we predict that the impact of CEO’s environmental awareness on technological innovation is more evident in private enterprises with strong monitoring.

We divided the samples into private enterprises and state-owned enterprises, which include state-owned enterprises and collective enterprises according to whether the actual controllers are private. Then we analyzed the samples through the regression analysis according to Model (1), and obtained the results as shown in Table 5.

When the dependent variable is R&D_t+1_, the Environment coefficient of private enterprise sample is positive and significant at the level of 1%, while that of state-owned enterprise sample is negative, but not significant. It indicates that the positive impact of the CEO’s environmental awareness on R&D investment is more obvious in private enterprises. When the dependent variable is Patent1_t+1_, the Environment coefficients of the samples of private enterprises and state-owned enterprises are both positive and significant at the level of 1%, but the Environment coefficients of the samples of private enterprises are slightly larger than those of the samples of state-owned enterprises (0.136 > 0.096). Therefore, the positive impact of CEO’s environmental awareness on R&D output is slightly stronger in private enterprises than those in state-owned enterprises. To test whether ownership type will moderate the impact of CEO’s environmental awareness on R&D efficiency, we control R&D investment on the right side of the equation and use Patent1_t+1_ as a dependent variable. According to the regression results, we find that the Environment coefficients of the samples of private enterprises and state-owned enterprises are still positive, but the Environment coefficients of private enterprises are significantly higher than those of state-owned enterprises, and the coefficients value of private enterprises are also greater than those of state-owned enterprises. Therefore, the positive impact of CEO’s environmental awareness on R&D efficiency is stronger in private enterprises than those in state-owned enterprises. Therefore, we can draw the following conclusion from the regression results of Table 5: the positive impact of CEO’s environmental awareness on enterprise technological innovation is more obvious in private enterprises with stronger monitoring.

#### 3.3.2. Institutional Investor Shareholding Ratio

Institutional investors have sufficient incentives to actively regulate listed companies in order to achieve returns on investment because of their large investment scale and limited selling freedom. Relevant studies have shown that institutional investors with a higher proportion of shareholdings are more likely to actively participate in corporate governance, thus making the external monitoring of enterprises relatively strong. According to the H2, we predict that the impact of CEO’s environmental awareness on technological innovation is more obvious in enterprises with a large proportion of institutional investors.

To test the hypothesis, we divide the sample enterprises into two groups according to the size of the institutional investors’ shareholding ratio. We then analyze the sample data through the regression analysis according to Model (1). The results are shown in Table 6.

When the dependent variable is R&D_t+1_, the Environment coefficient is positive and significant at the level of 5% for the sample with a higher proportion of institutional investors, while the Environment coefficient for the sample with a lower proportion of institutional investors is also positive, but it is not significant. Hence, the positive impact of CEO’s environmental awareness on R&D investment is more evident in enterprises with a higher proportion of institutional investors. When the dependent variable is Patent1_t+1_, the Environment coefficients of the samples with lower institutional investors and higher institutional investors are both positive, but that of the sample with higher institutional investors is more significant. Therefore, the positive impact of CEO’s environmental awareness on R&D output is significant in enterprises with high institutional investors’ shareholding. In order to test whether institutional investors’ ownership ratio can regulate the impact of CEO’s environmental awareness on R&D efficiency, we control R&D investment on the right side of the equation and treat Patent1_t+1_ as a dependent variable. According to the regression results, we find that the Environment coefficients of the two groups are positive, but the Environment coefficients of the samples with the higher proportion of institutional investors are significant at the level of 1%, while the Environment coefficients of the samples with the lower proportion of institutional investors are not significant. Therefore, the positive impact of CEO’s environmental awareness on R&D efficiency is greater in enterprises with a higher proportion of institutional investors. Thus, we can draw a conclusion from the regression results of Table 6: the positive influence of CEO’s environmental awareness on technological innovation is more obvious in the enterprises with higher proportion of institutional investors.

#### 3.3.3. Analyst Coverage

Analysts have corporate governance function, can effectively motivate managers, and improve a CEO’s investment decisions. The more analysts there are, the greater the external regulatory pressure and the lower the probability of violation. According to H2, we predict that the impact of CEO’s environmental awareness on technological innovation will be more evident in enterprises with more analysts.

To test the hypothesis, we divide the sample firms into two groups according to the analyst coverage. We then regress the two sets of observational reference models (1) and derive the results shown in Table 7.

When the dependent variable is R&D_t+1_, the Edu coefficient of the sample with higher analyst coverage is positive and significant at the level of 5%, while the Environment coefficient of the sample with lower analyst coverage is also positive, but it is not significant. Therefore, the positive impact of CEO’s environmental awareness on R&D investment is more evident in enterprises with higher analyst coverage. When the dependent variable is Patent1_t+1_, the Environment coefficients of the samples with lower analyst coverage and those with higher analyst coverage are both positive, but the samples with higher analyst coverage have larger coefficients. Therefore, the positive impact of CEO’s environmental awareness on R&D output is more evident in enterprises with higher analyst coverage. So as to test whether the analyst average can moderate the impact of CEO’s environmental awareness on R&D efficiency, we control R&D investment on the right side of the equation and use Patent1_t+1_ as a dependent variable. According to the regression results, we find that the Environment coefficients of both groups are positive, but the higher analyst coverage, the greater the value of the Environment coefficients. Therefore, the positive impact of CEO’s environmental awareness on R&D efficiency is significant in the higher analyst coverage. Therefore, we can conclude that the positive impact of CEO’s environmental awareness on enterprise technological innovation is more obvious in enterprises with a higher analyst coverage.

### 3.4. Robustness Check

#### 3.4.1. System GMM Estimation Test

Considering that the influence of CEO environmental awareness on enterprise technology innovation may have endogenous problems, we establish a dynamic panel model and use the system GMM estimation method to check whether the results are stable. System GMM estimates rely primarily on two tests to verify their applicability. The first is the Hansen test, which is used to test whether the tool variables are accurate in the case of over-identification, and the original assumption is that the tool variables are correct. The second is the sequence correlation test, we will check whether the error term after the difference is a second-order sequence correlation. From the AR (2) and Hansen J test results of Table 8, it can be seen that the GMM estimation method is suitable, and the Sargan test results of the three models are all greater than 0.1, indicating that there is no endogeneity problem. Specifically, the lags in R&D investment, patent output, and innovation efficiency are highly significant, indicating that the company’s technological innovation is sustainable. Under the system GMM estimation method, the influence of the CEO’s environmental awareness on the three explained variables is still significantly positive, indicating that the positive correlation between the CEO’s environmental awareness and enterprise technology innovation is robust. Hence, the hypothesis H1 is supported again.

#### 3.4.2. Deleting Firms with No Information on R&D Expenditures

We collect plenty of R&D input data manually since China’s financial disclosure system does not require listed companies to disclose R&D input data. In order to eliminate the possibility of misleading results, we eliminate the observation values without R&D input information, and then regress the remaining 3034 observations according to Model 1 again. The regression results are shown in Table 9. Environment coefficients in (1), (2), (3) of the table are all positive, and all pass the significant test. It shows that the CEO’s environmental awareness is still positively correlated with R&D input, R&D output and R&D efficiency after deleting the observations without information on R&D input. Therefore, we further confirm the hypothesis H1 that CEO’s environmental awareness can significantly promote technological innovation.

#### 3.4.3. Other Patent Variables

In order to ensure that our conclusions are more reliable, we replace the number of invention patents with the number of invention patents issued. For H1b and H1c, we regress Model 1 again and get the regression results as shown in Table 10. Environment coefficients in (1) and (2) of the table are both positive and significant at the level of 1%. This indicates that CEO’s environmental awareness is still positively correlated with R&D output and R&D efficiency after replacing the R&D output indicators, which is consistent with the test results of H1b and H1c.

## 4. Conclusions

### 4.1. Key Findings

This paper studies the impact of CEO’s environmental awareness on technological innovation. Based on a large sample of the Chinese A-share listed firms during the years between 2003 and 2014, our empirical studies and results reach the following conclusions. Firstly, a CEO’s environmental awareness can significantly promote the technological innovation of his/her enterprise. CEO who are more environmentally conscious invest more in R&D activities, obtain more patents and achieve greater innovative success. The reasons are the following. CEOs with strong environmental awareness can clearly understand the benefits and risks associated with implementing green innovation, hence their subjective innovation will be stronger. At the same time, CEOs with high environmental awareness can focus on the attention from stakeholders on green innovation, actively develop new products to meet the needs of stakeholders, and thus improve the efficiency of enterprise technological innovation. Secondly, the positive effect of CEO’s environmental awareness on technological innovation is more evident in an environment with strong monitoring. Specifically, the promotional effect of CEO’s environmental awareness on technological innovation is more pronounced in private enterprises, enterprises with a large proportion of institutional investors, and enterprises with large analyst coverage. Thirdly, we confirm the robustness of our main model and results after excluding the samples with no information on R&D input and after employing other possible patent indicators.

### 4.2. Theoretical Implications

This article enriches the existing literature in the following two aspects. Firstly, we find that CEO’s environmental awareness can significantly promote the technological innovation of his/her enterprise. This is consistent with the results of Egri and Herman [18] and Burki and Dahlstorm [19]. However, at the same time, it supplements the relationship between CEO environmental awareness and non-environmental technology innovation, enriching the content of Upper Echelon Theory and Technological Innovation Theory. Secondly, we also find that the positive effect of CEO’s environmental awareness on technological innovation is more evident in an environment with strong monitoring. This is consistent with several existing studies such as Jensen and Meckling [24], Knyazeva [25], Frank and li [26], Kochhar and David [27], and Jensen [28]. As Kim [29] emphasizes, external regulators such as institutional investors or analysts can participate in corporate governance through their own expertise and information resources. They can also motivate CEOs to carry out technological innovation and focus on long-term research and development results. From this perspective, this paper identifies a mechanism of monitoring exerting on technological innovation, which is to stimulate the internal innovation potential of CEOs with high level of environmental awareness to promote the growth of enterprise technology innovation.

### 4.3. Managerial and Policy Implications

Technological innovation can enhance the core competitive edge of enterprises. Based on our empirical analysis and results, we put forward the following policy recommendations with the goal of improving the level of technological innovation and providing managerial insights for developing country enterprises to implement green innovation strategy. Firstly, the government should help cultivate CEOs’ awareness of environmental protection, initiate training courses to enhance CEO’s understanding of the benefits and risks of green innovation, and introduce relevant policies such as tax incentives and subsidies to encourage CEO to implement environmental protection innovation. Secondly, the government should strengthen the monitoring of state-owned enterprises and deal with the principal-agent problem. This can encourage CEOs with higher environmental awareness to engage in environmental protection innovation activities, and actively promote technological innovation of their enterprises. Thirdly, the government should actively promote the development of institutional investors and analysts, improve the structure of institutional investors and encourage institutional investors and analysts to participate in corporate governance. This can allow for them to play better monitoring roles which can help CEOs achieve effective R&D innovation.

### 4.4. Limitations and Future Research Directions

This research empirically examines the influence of CEO’s environmental awareness on enterprise technological innovation and the regulatory effect of monitoring on the relationship between them. However, the relationship between CEO’s environmental awareness and green technological innovation needs further study. Furthermore, case studies should be conducted to test the possible causal relationship because it is difficult to clearly define and observe the input and output of an enterprise’s green R&D. Moreover, due to the data availability limitation, in this research, we collect and analyze sample data from Chinese listed companies during the years between 2003 and 2014 only. In future research, more sample data from other years and/or other countries can be collected and analyzed to further check the robustness of our results. In addition, the relationship between environmental awareness of other executives or employees and technological innovation is also worth exploring in the future.

## Figures and Tables

**Table 1:** Variable definitions.

Variables	Definitions
**Dependent variables**	
R&D	The R&D expenditures divided by lagged total assets.
Patent1	The log of one plus the number of patent applications.
Patent2	The log of one plus the number of patent grant.
**CEO’s awareness of environmental protection**	
Environment	1 means that only one of the three sub-indexes is ”1”, 2 means that two of the three sub indicators are “1”, 3 means that all three sub-indexes are “1”.
**Controls**	
Sales	The log of sales.
PPE	The ratio of PPE (Property, plant, and equipment) over the total assets.
Return	Stock return.
Size	The log of the market value of equity
MTB	The market value of equity divided by the book value of equity
Sales Growth	Sales growth
ROA	The income before extraordinary items divided by lagged total assets.
Leverage	The book value of total liabilities divided by lagged total assets.
CFO	The net cash flow from operating activities divided by lagged total assets.
**Moderating variable**	
State	A dummy variable that equals 1 if the firm is Privately-owned and 0 otherwise.
Ins	A dummy variable that equals 1 if the percentage of shares held by institutional investors above median and 0 otherwise.
Analyst	A dummy variable that equals 1 if the number of analysts following the firm above median and 0 otherwise.

**Table 2:** Descriptive statistics.

Variables	High Environmental Awareness CEO (N = 2209)			Low Environmental Awareness CEO (N = 5406)		
	Mean	Median	Std.dev	Mean	Median	Std.dev
**Dependent variables**						
R&D_t_	0.668	0.000	1.130	0.593	0.000	1.123
Patent1_t_	0.570	0.000	1.019	0.567	0.000	1.059
Patent2_t_	0.305	0.000	0.700	0.366	0.000	0.795
**Controls**						
Sales_t_	20.928	20.827	1.425	20.804	20.747	1.298
PPE_t_	0.266	0.130	0.175	0.253	0.221	0.175
Return_t_	0.160	-0.029	0.658	0.222	0.001	0.701
Size_t_	15.034	14.901	0.956	14.968	14.878	0.948
MTB_t_	3.314	1.755	2.746	3.384	2.635	2.776
SalesGrowth_t_	0.221	0.145	0.504	0.242	0.163	0.508
ROA_t_	0.038	0.038	0.060	0.037	0.066	0.062
Leverage_t_	0.434	0.434	0.213	0.452	0.465	0.201
CFO_t_	0.045	0.045	0.078	0.044	0.044	0.082
**Moderating variable**						
State	0.500	0.000	0.500	0.499	1.000	0.500
Ins	0.509	0.000	0.500	0.496	1.000	0.500
Analyst	0.421	0.000	0.492	0.408	0.000	0.494

**Table 3:** Pearson correlation test.

	R&D_t+1_	Patent1_t+1_	Patent2_t+1_	Env	Sales_t_	PPE_t_	Return_t_	Size_t_	MTB_t_	SalesGrowth_t_	ROA_t_	Leverage_t_	CFO_t_
R&D_t+1_	1												
Patent1_t+1_	0.352 ***	1											
Patent2_t+1_	0.148 ***	0.641 ***	1										
Env	0.015	0.025 **	0.051 ***	1									
Sales_t_	0.092 ***	0.167 ***	0.181 ***	0.018	1								
PPE_t_	−0.201 ***	−0.128 ***	−0.051 ***	−0.043 ***	0.172 ***	1							
Return_t_	0.012	−0.014	0.037 ***	0.039 ***	−0.018	0.005	1						
Size_t_	0.025 **	0.237 ***	0.217 ***	−0.014	0.657 ***	0.029 **	0.229 ***	1					
MTB_t_	0.041 ***	−0.025 **	−0.003	0.024 **	−0.262 ***	−0.096 ***	0.017	0.038 ***	1				
SalesGrowth_t_	−0.018	−0.014	0.011	0.021 *	0.104 ***	−0.029 **	0.075 ***	0.113 ***	0.067 ***	1			
ROA_t_	0.189 ***	0.146 ***	0.130 ***	−0.017	0.163 ***	−0.133 ***	0.128 ***	0.390 ***	−0.018	0.208 ***	1		
Leverage_t_	−0.330 ***	−0.116 ***	−0.0180	0.045 ***	0.381 ***	0.165 ***	0.015	0.008	0.094 ***	0.066 ***	−0.379 ***	1	
CFOt	0.012	0.0110	0.037 ***	−0.015	0.156 ***	0.268 ***	0.130 ***	0.201 ***	0.001	0.048 ***	0.340 ***	−0.101 ***	1

* Statistical significance at the 10% level. ** Statistical significance at the 5% level. *** Statistical significance at the 1% level.

**Table 4:** CEOs’ environmental awareness and firms’ innovation.

Dependent Variables	R&D_t+1_	Patent1_t+1_	Patent1_t+1_
	(1)	(2)	(3)
Environment_t_	0.068 **	0.124 ***	0.112 ***
	(2.24)	(2.59)	(2.61)
R&D_t_			0.204 ***
Sales_t_	0.035 **	0.004	−0.002
	(2.15)	(0.16)	(−0.09)
PPE_t_	−0.340 ***	−0.420 ***	−0.367 ***
	(−4.42)	(−3.30)	(−3.00)
Return_t_	0.163 ***	−0.057 **	−0.085 ***
	(6.55)	(−2.32)	(−3.49)
Size_t_	−0.153 ***	0.229 ***	0.258 ***
	(−6.99)	(5.31)	(6.01)
MTB_t_	0.023 ***	−0.013 **	−0.018 ***
	(4.68)	(−2.25)	(−3.15)
Sales Growth_t_	−0.001	−0.036 *	−0.033
	(−0.02)	(−1.76)	(−1.64)
ROA_t_	1.474 ***	0.362	0.119
	(6.41)	(1.32)	(0.46)
Leverage_t_	−0.924 ***	0.067	0.237 **
	(−11.91)	(0.56)	(2.00)
CFO_t_	0.535 ***	0.146	0.049
	(3.41)	(0.88)	(0.30)
Number of observations	7615	7615	7615
Adjusted R-squared	0.324	0.205	0.235
F	111.56 ***	24.52 ***	25.48 ***

This table presents regression estimation of the impact of CEO’s environmental awareness on firm’s innovation. The values of all continuous variables are Winsorized mean at 1% and 99%, and the standard deviation in regression is concentrated in enterprises. T-Statistics are reported in parentheses. All variables are defined in Table 1. * Statistical significance at the 10% level. ** Statistical significance at the 5% level. *** Statistical significance at the 1% level.

**Table 5:** The moderating effect of ownership type.

Dependent Variables	R&D_t+1_	R&D_t+1_	Patent1_t+1_	Patent1_t+1_	Patent1_t+1_	Patent1_t+1_
	(1)	(2)	(3)	(4)	(5)	(6)
	State-owned	Privately-owned	State-owned	Privately-owned	State-owned	Privately-owned
Environment_t_	−0.052	0.192 ***	0.096 **	0.136 **	0.108 **	0.144 ***
	(−1.01)	(2.89)	(2.14)	(2.25)	(2.39)	(3.03)
R&D_t_					0.285 ***	0.169 ***
					(5.18)	(11.29)
Number of observations	3803	3812	3803	3812	3803	3812
Adjusted R-squared	0.242	0.306	0.205	0.198	0.241	0.224
F	7.39 ***	33.78 ***	8.32 ***	16.84 ***	8.90 ***	33.50 ***

The other control variables are consistent with Table 3. * Statistical significance at the 10% level. ** Statistical significance at the 5% level. *** Statistical significance at the 1% level.

**Table 6:** The moderating effect of institutional shareholding.

Dependent Variables	R&D_t+1_	R&D_t+1_	Patent1_t+1_	Patent1_t+1_	Patent1_t+1_	Patent1_t+1_
	(1)	(2)	(3)	(4)	(5)	(6)
	Low	High	Low	High	Low	High
Environment_t_	0.032	0.098 **	0.082 *	0.12 ***	0.104	0.112 ***
	(0.94)	(2.45)	(1.84)	(2.96)	(1.63)	(2.88)
R&D_t_					0.206 ***	0.193 ***
					(5.87)	(7.13)
Number of observations	3807	3808	3807	3808	3807	3808
Adjusted R-squared	0.274	0.393	0.191	0.214	0.220	0.243
F	75.60 ***	44.32 ***	18.32 ***	15.29 ***	18.88 ***	15.70 ***

The other control variables are consistent with Table 3. * Statistical significance at the 10% level. ** Statistical significance at the 5% level. *** Statistical significance at the 1% level.

**Table 7:** The moderating effect of analyst.

Dependent Variables	R&D_t+1_	R&D_t+1_	Patent1_t+1_	Patent1_t+1_	Patent1_t+1_	Patent1_t+1_
	(1)	(2)	(3)	(4)	(5)	(6)
	Low	High	Low	High	Low	High
Environment_t_	0.05	0.096 **	0.096 **	0.166 **	0.088 **	0.148 **
	(1.60)	(2.13)	(2.11)	(2.17)	(2.03)	(1.96)
R&D_t_					0.197 ***	0.203 ***
					(5.92)	(5.34)
Number of observations	4479	3136	4479	3136	4479	3136
Adjusted R-squared	0.368	0.274	0.215	0.195	0.242	0.225
F	36.63 ***	79.83 ***	12.65 ***	15.16 ***	13.29 ***	15.63 ***

The other control variables are consistent with Table 3. * Statistical significance at the 10% level. ** Statistical significance at the 5% level. *** Statistical significance at the 1% level.

**Table 8:** System GMM estimation results.

Dependent Variables	R&D_t+1_	Patent1_t+1_	Patent1_t+1_
	(1)	(2)	(3)
R&D_t_	0.255 ***		0.045 *
	(0.045)		(0.026)
Patent1_t_		0.521 ***	0.510 ***
		(0.079)	(0.079)
Environment_t_	0.020 **	0.016 *	0.020 **
	(0.023)	(0.041)	(0.041)
Sargan test	0.205	0.361	0.429
Hansen J test	0.844	0.127	0.132
AR (2) test	0.351	0.109	0.127
chi2	581.94	1110.67	1109.61
Number of observations	3685	3685	3685

The other control variables are consistent with Table 3. In parentheses is the standard deviation of each statistic. * Statistical significance at the 10% level. ** Statistical significance at the 5% level. *** Statistical significance at the 1% level.

**Table 9:** Firms with R&D investment information.

Dependent Variables	R&D_t+1_	Patent1_t+1_	Patent1_t+1_
	(1)	(2)	(3)
Environment_t_	0.214 ***	0.156 **	0.114 **
	(4.22)	(2.05)	(2.35)
R&D_t_			0.194 ***
			(10.59)
Number of observations	3034	3034	3034
Adjusted R-squared	0.239	0.133	0.164
F	111.56 ***	24.52 ***	69.73 ***

The other control variables are consistent with Table 3. * Statistical significance at the 10% level. ** Statistical significance at the 5% level. *** Statistical significance at the 1% level.

**Table 10:** Other patent variables.

Dependent Variables	Patent2_t+1_	Patent2_t+1_
	(1)	(2)
Environment_t_	0.104 ***	0.1 ***
	(3.30)	(3.20)
R&D_t_		0.080 ***
		(5.03)
Number of observations	7615	7615
Adjusted R-squared	0.137	0.146
F	69.73 ***	13.68 ***

The other control variables are consistent with Table 3. * Statistical significance at the 10% level. ** Statistical significance at the 5% level. *** Statistical significance at the 1% level.
